# Written surgical informed consent elements in pediatric differences of sex development: pediatric urologist and endocrinologist perspectives

**DOI:** 10.3389/fruro.2023.1188822

**Published:** 2023-07-10

**Authors:** Zoe K. Lapham, Melissa Gardner, Sydney Sheinker, Kristina I. Suorsa-Johnson, Barry A. Kogan, Peter A. Lee, David E. Sandberg

**Affiliations:** ^1^ Susan B. Meister Child Health Evaluation and Research Center, Department of Pediatrics, University of Michigan Medical School, Ann Arbor, MI, United States; ^2^ College of Literature, Science, and the Arts, University of Michigan, Ann Arbor, MI, United States; ^3^ Department of Pediatrics, University of Utah Spencer Fox Eccles School of Medicine, Salt Lake City, UT, United States; ^4^ Departments of Urology and Pediatrics, Division of Urology, Albany Medical College, Albany, NY, United States; ^5^ Department of Pediatrics, Division of Endocrinology, Penn State College of Medicine, Hershey, PA, United States; ^6^ Division of Pediatric Psychology, Department of Pediatrics, University of Michigan Medical School, Ann Arbor, MI, United States

**Keywords:** differences of sex development (DSD), disorders of sex development (DSD), intersex, informed consent, pediatric, genital surgery

## Abstract

**Introduction:**

Elective aspects of surgical management of pediatric differences of sex development (DSD) are associated with controversy. We examined North American pediatric urologist and endocrinologist perspectives regarding recommended and existing informed consent elements for written consent documents prior to pediatric genital surgery.

**Methods:**

Focus groups with pediatric urologist and endocrinologist members of the Societies for Pediatric Urology (SPU, n=8) or Pediatric Endocrine Society (PES, n=8) were held to identify elements of informed consent for DSD-related urogenital surgery. Elements were subsequently included in web-based surveys in 2003 and 2020 (SPU: n=121 and 143; PES: n=287 and 111, respectively). Participants rated their level of agreement with including each element in informed consent documents. In 2020, participants reported whether documents they use in clinical practice incorporate these elements.

**Results:**

Focus groups identified four elements of informed consent: on-going debate over pediatric genital surgery; potential needs for multiple procedures; possible gender change and surgical reversal; and non-surgical alternatives. Across both years and both specialties, a majority (79% to 98%) endorsed the four elements, with significant between-group differences. Significantly more PES than SPU participants reported not knowing whether specific elements were included in current written informed consent; of those who knew, the majority (66% to 91%) reported inclusion.

**Discussion:**

Specialists agree with including these four elements in written informed consent documents. Endocrinologists are not always familiar with the exact elements included. The degree to which non-surgeon members of the care team should be involved in the written informed consent process is an open question.

## 1 Introduction

Informed consent is fundamental to healthcare decisions (1); it involves both a process and an outcome. The informed consent process includes three essential components: 1) relaying the relevant information (i.e., the nature of the procedure, risks, benefits, and alternatives); 2) assessing patient and/or surrogate understanding and providing time for questions; while 3) ensuring agreement with the plan is voluntary (2–5). The outcome of this process often takes the form of a written record, signed by patient and provider, that documents elements of the process and the final agreed-upon course of action.

In North America, there are both legal and ethical aspects of informed consent. Legally, informed consent can vary by jurisdiction, with some formally codifying legal informed consent (6, 7). Typically, failure to acquire informed consent for a medical procedure is deemed negligent in court proceedings (6). Legal best practice recommendations include that informed consent for, or refusal of, a specific procedure, whether oral or written, is to be noted in the medical record (8). Ethical considerations for informed consent are based in the concept of autonomy: individuals have the right to make decisions based on their own beliefs, knowledge, and reasoning (9). For a choice to be autonomous, it must be voluntary, informed, and made by an individual with the capacity to make decisions (9).

Special considerations for informed consent apply to certain areas of medicine. In pediatrics, parental permission, with child assent as is developmentally appropriate, serves as informed consent. Aligned with the belief that parents are typically best positioned to make healthcare decisions on behalf of their child’s best interests, the American Academy of Pediatrics states, in most cases, physicians have an ethical and legal obligation to obtain parental permission (10); this officially positions the caregivers as essential stakeholders to the informed decision-making and consent process. Parental decision-making inherently requires balancing both preserving the patient’s opportunity for future choices and protecting the child’s current well-being (11). Informed consent is also complicated when long-term outcomes of healthcare decisions are not entirely clear. Most novel surgical procedures are developed with the best of intentions, but, for obvious reasons, long term outcome data are not available (12). It is essential that these limitations are conveyed to families during the informed consent process; written informed consent documents can be a way to standardize that communication based on consensus among experts, the existing literature, and the current unknowns about procedure outcomes.

Within pediatric differences of sex development^1^ (DSD), parents are asked to make decisions for their infant/young child that have uncertain long-term outcomes and limited data to support decision making. DSD are congenital conditions in which the development of the chromosomal, gonadal, or anatomic sex is atypical (13, 14). Differences of sex development are commonly identified in the newborn period because of either atypical genital appearance or discordance between prenatal testing (karyotype and/or imaging) and neonatal physical exam (13, 14). Rarely in DSD, urgent surgery is required to avert life-threatening circumstances or to prevent permanent disability (e.g., creating unobstructed outlets for urine or stool). Elective surgeries, including those intended to address genital function and appearance, may also form part of clinical management plans. Unlike urgent procedures, elective procedures in DSD are associated with controversy related to unresolved questions concerning the propriety of performing this type of procedure on infants and children before they are competent to participate in the informed consent process. Taking this conundrum into consideration, clinical practice guidelines generally recommend discussing all options with parents as part of a decision-making process rather than recommending a specific course of action (15, 16).

Some patient advocacy (17, 18) and human rights (19–23) organizations, as well as U.S. State legislators (24, 25) have called for a complete ban on early elective genital surgery. However, other patient support and advocacy organizations representing those born with a DSD emphasize the importance of parents having both the right and responsibility to make informed decisions about early surgery on behalf of their young child (26). Concerns regarding parental proxy decision-making include the extent to which parents are fully informed about the full range of options and the harms and benefits associated with each option; there are concerns that parents may need extensive education on this information to make fully informed decisions that goes beyond recommendations in clinical practice guidelines (27–30).

The aim of this study, a component of a larger project, is to explore the recommendations of pediatric urologists and endocrinologists – specialties routinely involved in the clinical management of DSD – regarding written informed consent in elective DSD genital surgery and to identify which elements are currently included in informed consent documents.

## 2 Methods

### 2.1 Surgical informed consent element identification

In the context of developing a survey of clinician recommendations for the management of complex or controversial topics in intersexuality/DSD (see **Supplementary Material**), focus groups were convened to identify themes pertinent to the investigation. Focus group members included junior and senior members of the Societies for Pediatric Urology (SPU, n=8) and the (Lawson-Wilkins) Pediatric Endocrine Society^2^ (PES, n=8) nominated for participation by colleagues who thought their opinions would be particularly informative; a geographically diverse sample within North America was sought; groups were convened by conference call.

### 2.2 Clinician survey

Survey items were generated based on focus group input and literature review. Items comprised five sections, including the *written surgical informed consent* section that is the focus of this manuscript. In addition to generating survey items, focus group participants recommended web-based administration to facilitate recruitment. A preliminary survey was pilot tested with a subgroup of focus group participants, with others checking for comprehensiveness of content coverage and survey response options.

The full survey was administered in 2003, 2010, and 2020 to SPU and PES membership. Eligibility criteria included current membership in either society, specialization in pediatric endocrinology or urology, current or prior history of providing care to patients with DSD, and practice location in North America (United States, Canada, Mexico). Exclusion criteria included being a survey co-investigator, participation in survey creation, and status as retired or emeritus. Survey development that included focus groups, pilot testing, followed by administration of the 2003 survey, were approved by the Institutional Review Board at the University at Buffalo School of Medicine and Biomedical Sciences. The University of Michigan Medical School IRBMed approved continued analysis of 2003 data under a federal exemption from continuing IRB review status. The 2010 survey administration was reviewed by the IRBMed and initially (2010) approved as human subjects research. Subsequently (2015), IRBMed determined that the study was exempt from further review. The 2020 survey administration was reviewed by IRBMed – which determined that the study was exempt from ongoing IRB review. In all years, participants were promised confidentiality.

This manuscript reports on four elements, identified by focus group members, comprising the *written surgical informed consent* section of the survey. This section was included in only the 2003 and 2020 administrations. In both years, the research team sought approval from leadership of both SPU and PES to survey their membership and to provide member rosters that include contact information. Society leadership approved the research and provided rosters, apart from PES in 2020 – citing concerns about participant burden. As such, in 2020, PES member recruitment was limited to only those who had been previously invited for participation in 2003 or 2010. In 2003, 408 (121 SPU; 287 PES) members participated; in 2020, 254 (143 SPU; 111 PES) participated. Additional sample characteristics are summarized in **Table 1**.

**Table 1:** Participant Demographics.

	2003				2020			
	SPU		PES		SPU		PES	
	n	%	n	%	n	%	n	%
Eligible invited participants	190	100	516	100	281	100	434	100
Completed survey	121	63.7	287	55.6	143	50.9	111	25.6
Began survey without completing	11	5.8	13	2.5	21	7.5	12	2.8
Declined participation^1^	10	5.3	48	9.3	5	1.8	5	1.2
No response	48	25.3	168	32.6	107	38.1	304	70.0
No contact information	0	0.0	0	0.0	5	1.8	2	0.5
Sex								
Male	115	95.0	172	59.9	122	85.3	58	52.3
Female	6	5.0	115	40.1	21	14.7	52	46.8
Other^2^	--	--	--	--	0	0.0	1	0.9
Practice Community								
Large Metropolitan	86	71.7	173	60.7	107	74.8	76	68.5
Small Metropolitan	33	27.5	107	37.5	34	23.8	34	30.6
Nonmetropolitan / Rural	1	0.8	5	1.8	2	1.4	1	0.9
Practice Country								
United States	114	94.2	269	93.7	137	95.8	103	92.8
Canada	7	5.8	18	6.3	6	4.2	8	7.2
Practice Setting								
Medical School or Hospital	78	65.5	212	76.8	104	72.7	94	84.7
Solo or Two-physician Practice	16	13.4	22	8.0	6	4.2	5	4.5
Group Practice	24	20.2	33	12.0	30	21.0	10	9.0
HMO	1	0.8	9	3.3	3	2.1	1	0.9
Other	0	0.0	0	0.0	0	0.0	1	0.9
	median	min-max	median	min-max	median	min-max	median	min-max
Year of Birth	1953	1932-1966	1952	1921-1971	1966	1932-1983	1960	1942-1977
Cases Seen Over Career	50	5-999	40	1-1,000	50	5-1,500	50	4-1,000

^1^ A common reason cited for declining participation was being “too busy”.

^2^ “Other” was included as a response option in 2020 only.

In both years of survey administration, participants were presented with the four elements and asked to rate whether each should be included on written informed consent documents using a Likert-type scale where 1 = strongly disagree, 2 = disagree, 3 = agree, and 4 = strongly agree. In the 2020 survey, participants were also asked if these elements were included in the consent documents that they (or members of their team) use.

### 2.3 Data analysis plan

Qualitative data, comprising four elements of written surgical informed consent identified by focus group members are presented verbatim. Descriptive statistics (e.g., frequencies, means) were calculated to characterize participant characteristics and level of agreement with each element. Quantitative data analysis was facilitated by IBM SPSS Statistics for Windows, Version 27.0 (31). χ2 analyses, coupled with two-tailed z-tests for independent proportions, using Bonferroni corrections were used to test for differences in responses between specialties (i.e., urologist *vs* endocrinologist) and over time (i.e., 2003 *vs* 2020).

Preliminary data analysis revealed few participants either “disagreed” or “strongly disagreed” with the inclusion of most elements; this resulted in the failure of expected cell counts to reach minimums required for χ2 analyses. As such, the “disagree” and “strongly disagree” response options were combined into a single category. Fisher’s Exact Test (32) was applied when expected cell counts continued to remain low after combining.

## 3 Results

### 3.1 Identified elements of surgical informed consent: 2002

Focus group members identified and arrived at consensus on four elements of written informed consent for genital surgery: 1) There is presently an on-going debate as to whether or not surgery is in the best interest of the child (“on-going debates”); 2) Genital abnormalities may take more than one procedure to correct and may in fact involve multiple procedures (“multiple procedures”); 3) In the future your child may have conflict with their assigned gender and therefore request further surgery to reverse the current surgery (“future gender change/surgical reversal”); and 4) The alternatives to the recommended surgical procedures have been fully explained to parents and they have been informed that one of these alternatives is to refuse surgical options altogether (“surgical alternatives”).

### 3.2 Clinician agreement with including identified elements in written informed consent: 2003 and 2020

Collapsing across time and specialty, the majority of participants either agreed or strongly agreed with the inclusion of each element in surgical written informed consent. Ranked from highest to lowest, participants most frequently agreed or strongly agreed with the inclusion of “multiple procedures” (646 observations, 97.6%), followed by “surgical alternatives” (642, 97.0%), “future gender change/surgical reversal” (534, 80.9%), and “on-going debates” (525, 79.3%).

**Figure 1:**
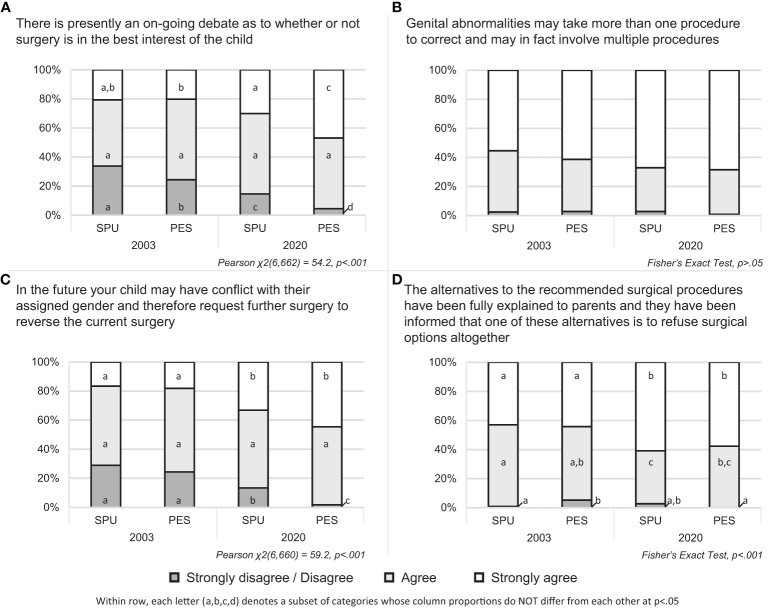
Agreement with including elements of written informed consent by provider specialty and year of administration.

Statistically significant differences were observed between specialty and over time for three elements: “on-going debates,” χ2 (6,662) = 54.2, p<.001; “future gender change/surgical reversal,” χ2(6,660) = 59.2, p<.001; and “surgical alternatives,” Fisher’s Exact Test, p<.001. Generally, higher levels of agreement were seen in 2020 than 2003 and among PES members compared with SPU members (**Figure 1**).

### 3.3 Presence of recommended elements in currently utilized consent documents: 2020

In 2020, participants from SPU and PES reported whether the four identified elements of informed consent were also included in their current informed consent documents. Most SPU members reported either personally performing genital surgery (n=123, 86%) or being a member of a team that offers genital surgeries for DSD (n=12, 8%). No PES members reported personally performing genital surgery; over half (n=73, 66%) reported being a member of a team that does so. Of those members who either perform surgery or are a member of a team that does, a minority of SPU (between 5% to 13% per element) and PES (38% to 47% per element) members reported they did not know if an element was present. For each element, significantly more PES members reporting not knowing than did SPU members (χ2, p<0.001).

Arranged from those most to least often reported as being present on the written informed consent documents they (or their team) use and adjusted to exclude those responding not knowing, the element reported as most often present in current written consent is “alternatives explained” (n=154, 91.1%) followed by “multiple procedures” (n=154, 89.5%), “future gender change and surgical reversal” (n=107, 68.2%), and “on-going debates” (n=112, 65.9%). Statistically significant differences between specialties were observed for “on-going debates,” χ2(1,7) = 12.2, p<.001 and “future gender change and surgical reversal” χ2(1,157) = 6.5, p<.05 (**Table 2**).

**Table 2:** Current inclusion of elements in written informed consent documents.

Element^1^	Societies for Pediatric Urology (SPU)				Pediatric Endocrine Society (PES)				χ2 (df,n)^2^	p-value
	Yes		No		Yes		No			
	n	%	n	%	n	%	n	%		
On-going debates	75	58.6	53	41.4	37	88.1	5	11.9	12.2 (1,70)	<.001
Multiple procedures	112	88.2	15	11.8	42	93.3	3	6.7	--	>.05
Future gender change & surgical reversal	74	62.7	44	37.3	33	84.6	6	15.4	6.5 (1,157)	<.05
Alternatives explained	113	90.4	12	9.6	41	93.2	3	6.8	--	>.05

^1^ The full text of each element is as follows: There is presently an on-going debate as to whether or not surgery is in the best interest of the child; Genital abnormalities may take more than one procedure to correct and may in fact involve multiple procedures; In the future your child may have conflict with their assigned gender and therefore request further surgery to reverse the current surgery; The alternatives to the recommended surgical procedures have been fully explained to parents and they have been informed that one of these alternatives is to refuse surgical options altogether.

^2^ Fisher’s Exact Test was substituted for Pearson χ2 in analyses where at least one cell had an expected count less than 5.

Percentage calculations are limited to those responding “yes” or “no”; those responding “I don’t know” were excluded.

## 4 Discussion

Most pediatric urologist and endocrinologist participants agreed with the inclusion of each of four elements proposed by their colleagues within written consent documents. Evidence for increased endorsement of elements was seen in both professional societies over time. Further, respondents noted that these elements were typically, but not always, present in informed consent documents. In contrast, current literature suggests that written informed consent documents tend to be generic and emphasize preventing litigation, rather than providing procedure-specific information (33). As a process, informed consent involves informing the patient’s caregivers about the relevant information required to provide informed consent; the written informed consent document constitutes the final step of that process. It is possible that participants employed a broad definition of what constitutes an element’s inclusion when considering whether an element is included in current informed consent documents. For example, participants may have interpreted that discussion of a specific statement (e.g., genital abnormalities may take more than one procedure to correct and may in fact involve multiple procedures) during the informed consent process was adequately represented by a more general written statement (e.g., potential harms and benefits of the proposed intervention have been discussed) in the actual document and (erroneously) responded “yes” that the element was included in their consent form.

Regarding between-specialty differences, significantly fewer participating SPU members reported “on-going debates” and “possible future gender change/reversal of surgery” were included in informed consent forms than PES members. Exactly why this occurred is unclear but may be related to differences between specialties regarding the level of detail different specialists believe is necessary to adequately consider an element as included in the written document. Members of the PES more frequently reported “I don’t know” regarding whether or not elements were included in their informed consent documents. Given no PES members reported performing early genital surgery, this finding may reflect that physicians who do not perform surgery are not involved in the written informed consent process. Whether all clinicians on a care team should be involved in all aspects of the informed consent process and documentation is an open question; team care is recommended for the DSD, but specifics about the level of involvement in the informed consent process and documentation are not specified in the most recent consensus statements for the field (14).

Strengths of the study include the initial generation of DSD-specific informed consent elements and their endorsement in 2003 and 2020 by members of two societies with expertise in the clinical management of DSD. Study participants’ reported experience reflected this; across years and specialties, average number of cases seen in career ranged between 66 and 105. By recruiting members of SPU and PES members for the focus that generated the elements, the working of items for the field survey likely reflect the language that these specialists might consider adopting in informed consent documents. An additional study strength was the relatively large sample size participating in the field survey in both 2003 and 2020. Although responses of “strongly disagree” and “disagree” needed to be combined to support inferential statistical analyses, this was not due a lack of statistical power to detect differences; instead, this reflects a level of consensus among clinicians that few disagreed with inclusion of the proposed elements. Finally, this survey was confidential, providing clinicians the assurance that their individual responses would not be available outside the study team.

A potential limitation of this study is that included informed consent elements proposed by focus group members when the group convened in 2002 may differ from elements that could be generated today. The four elements are not thought to be exhaustive of all candidates for informed consent prior to DSD-related genital surgery. Participant recruitment was limited to pediatric urologist and endocrinologist members of the SPU or PES; generalizability to other healthcare specialties within DSD care teams is unknown. Notably, pediatric and adolescent gynecologists, who are increasingly included in DSD care teams and perform DSD surgery (see [(34–44)] for a sample of DSD teams that include gynecology as part of their services) are not represented. In 2020, recruitment of PES members was limited to those who had been invited for participation to the larger clinician survey in 2003 or 2010. As such, PES members had been in practice for at least ten years at the time of the second survey on informed consent; views of newer members, reasonably anticipated to be younger endocrinologists who completed their training more recently, may not be adequately represented.

Additional factors that may have impacted the findings include those that may affect all survey research, including social desirability effects and self-selection bias. Opponents to early urogenital surgery have suggested that caregivers’ emotional distress surrounding the birth of a child with atypical genitalia (45–48) makes them predisposed to accepting presentations of surgery as beneficial (30). Some intersex advocates have also called for bans on early elective surgery in DSD, leading to proposed legislation in several states and two children’s hospitals in the U.S. limiting specific elective genital surgeries in DSD prior to when the patient can be meaningfully involved with decision-making (24, 25). The degree to which controversy in the field may affect responses – including participant self-selection into the survey – is unknown and could result in findings that are less generalizable. Finally, given both survey development and administration occurred in North America, generalizations beyond this region must be made with caution.

The informed consent process should consider including greater detail about harms, benefits, alternatives, and the nature of proposed procedures than is routinely the case in informed consent documents signed prior to surgical procedures currently performed as part of DSD care. Decisional regret (i.e., feelings of distress or remorse over a decision, linked to poor outcomes) has been identified in some caregivers following early surgery in DSD (49–51). More robust informed consent processes and documentation are expected to increase caregiver knowledge, thus reducing decisional regret (49–51). Documentation of the informed decision-making process supports patient- and family-centered care goals in pediatrics by providing official documentation in the medical record that can be referenced by providers and all family members, including the patient when old enough to understand, in the future, which supports the AAP’s recommendation both to include patient family’s unique insights into the care plan and share information with all children on the management of their own health care (52). Clear documentation supports these goals by providing a standardized mechanism for all stakeholders in the patient’s care to be able to reference (and, in the case of the child as they age, learn about) the history of care management, which may be essential to future care. DSD care teams should consider to what degree all team members should be involved in the decision-making process and written documentation – both in terms of: 1) establishing the general and specific statements that should be shared with proxy decision makers and documented through the signing of the informed consent document and 2) the nature of involvement recommended for non-surgical DSD team members in the informed consent process.

In a break from general statements regarding anticipated harms and benefits characteristic of many surgical informed consent documents, pediatric urologists and endocrinologists proposed the inclusion of four new and DSD-specific elements of written informed consent concerning elective genital surgery for pediatric patients with DSD. When presented to a larger group of their colleagues, the substantial majority agreed with the inclusion of these specific elements in written informed consent documents – with increasing endorsement over time. The extent to which all members of clinical teams, including those not performing surgery, should be involved in and knowledgeable about both the consent process and documentation of informed consent is an open question.

## Data availability statement

The raw data supporting the conclusions of this article will be made available by the authors, without undue reservation.

## Ethics statement

The studies involving human participants were reviewed and approved by Institutional Review Board (IRB) at the University at Buffalo School of Medicine and Biomedical Sciences and IRBMed at the University of Michigan Medical School. Written informed consent for participation was not required for this study in accordance with the national legislation and the institutional requirements.

## Author contributions

MG, KS-J, BK, PL, and DS contributed to the conception or design of this work. All authors contributed to the acquisition, analysis, or interpretation of data. ZL and MG completed the original draft of this manuscript. All authors contributed to the article and approved the submitted version.

## Footnotes

1 The umbrella term “disorders of sex development,” established through the 2006 Consensus Statement on Intersex Conditions (13), has been rejected by some patient advocates who believe that the word “disorder” fosters stigma. As such, we have substituted “differences” for “disorders” which preserves the acronym DSD. Further, “intersex” is a term preferred by some, we opted to use DSD as an expression of person-first language.

2 The Lawson Wilkins Pediatric Endocrine Society was founded in 1972; in 2010 the name of the Society was changed to the Pediatric Endocrine Society (PES).
