# Supplementary material for: Written surgical informed consent elements in pediatric differences of sex development: pediatric urologist and endocrinologist perspectives
Source: Front Urol. 2023 Jul 10;3:1188822. doi: 10.3389/fruro.2023.1188822 (PMC12327251; doi:10.3389/fruro.2023.1188822)
Supplement: Supplementary file 1 [file DataSheet_1.pdf]

Supplementary Material

DSD Clinician Survey: Surgical Informed Consent Survey and Participant Profile

Summary

A survey of pediatric urologists and endocrinologists on clinical management practices regarding disorders (differences) of sex development / intersex conditions was administered at three timepoints. Participants were recruited from membership rosters of two North American-based professional societies: the Societies for Pediatric Urology and the (Lawson-Wilkins) Pediatric Endocrine Society. Members were sent invitations to complete the online survey at three timepoints: three years prior to publication of the 2006 "Consensus statement on management of intersex disorders," four years following it, and four years following publication of the 2016 Consensus statement update.

This document details survey development and member participation related to Surgical Informed Consent items that were included in the first and third administrations of the survey.

Survey Development

Initial Survey Development

To assess pediatric urologist and endocrinologist opinion on clinical management practices regarding intersex conditions a web-based survey was created in 2002. Provisional survey items were generated based on a literature review and feedback from focus groups conducted by conference call. Focus groups were convened to identify themes pertinent to the investigation and canvass opinion regarding optimal survey administration format. Focus group participants included 16 junior and senior members of the Societies for Pediatric Urology (SPU) and the (Lawson-Wilkins)[1] Pediatric Endocrine Society (PES). Members were nominated for participation by colleagues who thought their opinions would be particularly informative; a geographically diverse sample was sought. Web-based administration to facilitate recruitment was the consensus of focus group participants. A preliminary survey was pilot-tested with a subgroup of focus group members with other members checking for comprehensiveness of content coverage and survey response options.

The final version of the 2003 Intersex Survey was administered in 2003-2004 and comprised five sections: 1) *Case Presentations,* 2) *Factors Affecting Life Satisfaction*, 3) *Surgical Informed Consent*, 4) *Mental Health Services and the DSD Team*, and 5) *Demographics*.

[1] Founded in 1972, the Lawson Wilkins Pediatric Endocrine Society formally changed its name to the Pediatric Endocrine Society (PES) in 2010.

Subsequent Survey Iterations

The survey was administered in 2003-04, 2010-2011, and 2020. Survey items were edited over time. Use of earlier data collection and analyses guided later data collection.

When data collection spanned >1 year, the first year in which data collection occurred is used in the label. These are hereafter referred to as the 2003, 2010, and 2020 surveys.

Limited changes were made to terminology/item wording, order of presentation, and inclusion/exclusion from the survey. A guiding principle behind any change was to limit the extent of changes made to allow for direct comparisons across time.

Edits to Surgical Informed Consent and Demographic items over time:

- Terminology changed from Intersex to DSD. In 2003, *intersex* applied to the medical condition; it was replaced by *disorders of sex development* following the 2006 Consensus Statement; by 2020, use of the term *intersex* re-emerged, but carried a different connotation for some - applying more to an identity than a medical condition, per se. Additionally, by 2020 the word *disorder* was viewed negatively by some who then supported the term *difference of sex development*. Changes in wording were intended to maintain a focus on the same set of medical conditions, despite changes in vernacular used to describe these conditions.
    - 2003: “Intersex” and “Intersexuality”
    - 2010: “Disorder of Sex Development (DSD)”
    - 2020: “Disorders/differences of sex development (DSD)”
- Use of earlier data collection and analyses to guide later data collection
    - 2003
        - Began with case presentations
        - Ended with demographics
    - 2010
        - Began with demographics
        - Did not include surgical informed consent items
    - 2020
        - Began with introduction, which included:
            - Self-administered eligibility screen
            - Opt-out with pre-populated reasons for opting out
        - Demographics followed the introduction
            - For participant sex, an “other” option was included
        - Reintroduced surgical informed consent items
        - Added items assessing whether surgical informed consent items were present in participants’ institutions’ consent documents

Survey Components

Table 1. Surgical Informed Consent-focused survey components

Section	Contents: Major Components
**Introduction**	Overview of survey<br>Eligibility screener – 2020 only
**Demographics**	Clinical practice and demographic characteristics
**Surgical Informed Consent**	Recommended elements<br>Current inclusion of elements – 2020 only

Table 2. Surgical Informed Consent-focused survey component order

Section	2003	2010	2020
1	Introduction	Introduction	Introduction
2	Surgical Informed Consent	Demographics	Demographics
3	Demographics	--	Surgical Informed Consent

Figure 1. Branching and skip logic used in survey administration

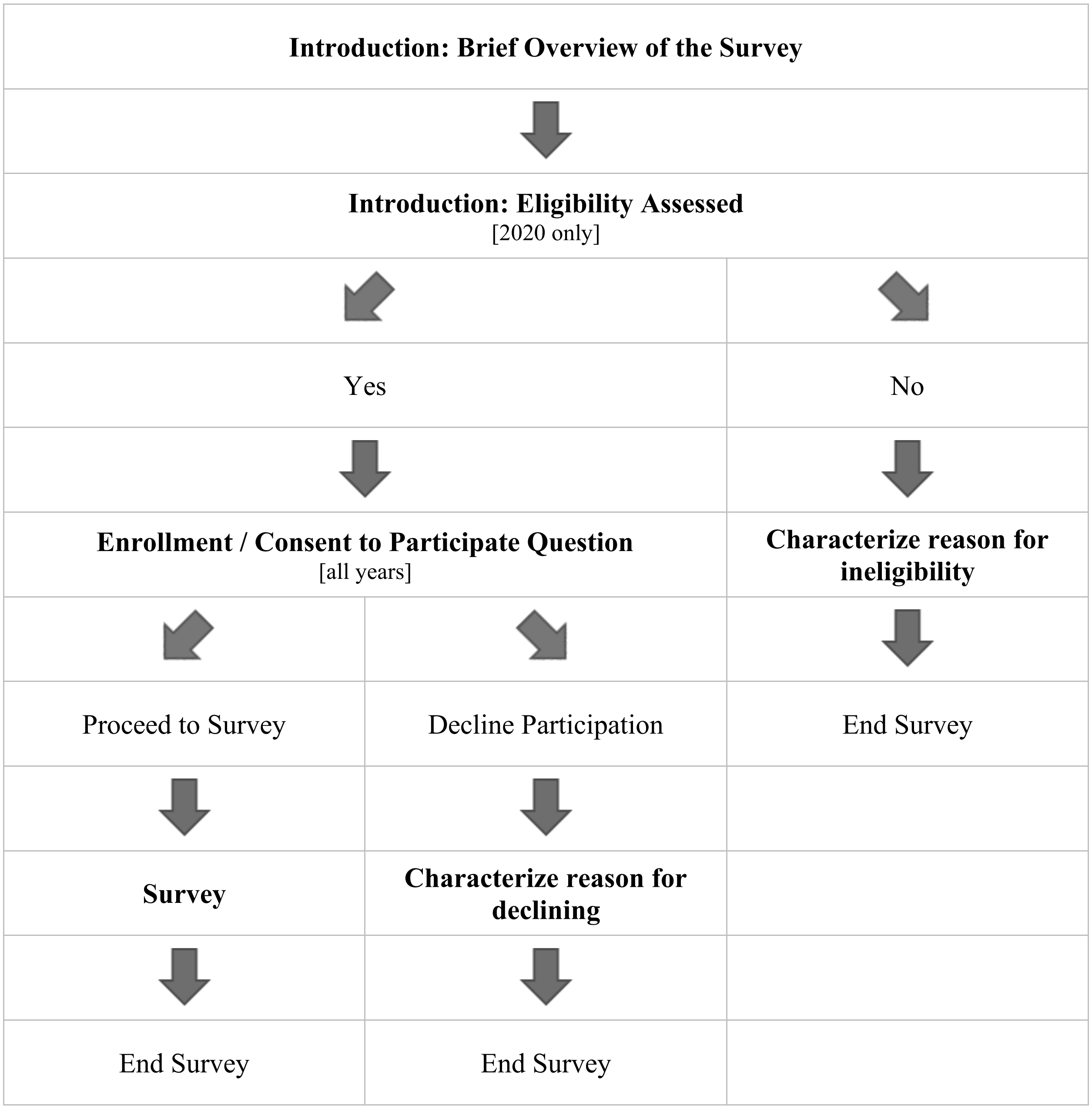

Figure 2: Branching and skip logic used in the surgical informed consent section

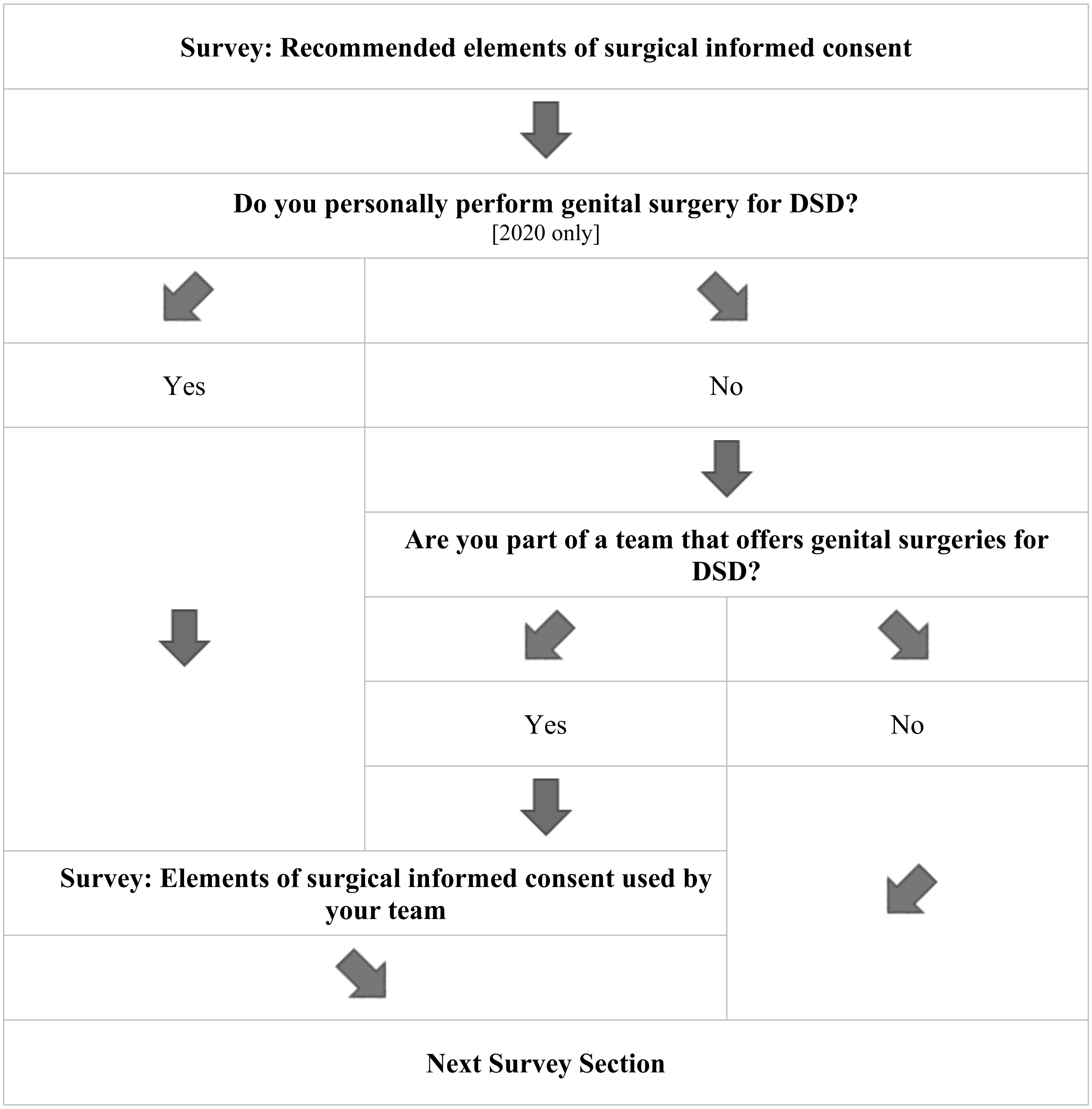

Survey Items

Notes: This survey was administered online; branching and skip logic were employed. Instances of branching and skip logic are shown below, with logic described in bracketed sections. Additional changes specific to one or more years are also noted in bracketed sections. Information shown in brackets was not visible to participants. The order of items presented below reflects that used in the 2020 survey.

Introduction: Eligibility [2020 only]

You were selected to complete this survey due to membership in the [relevant society name is displayed].

Disorders/differences of sex development (DSD) are “congenital conditions in which development of chromosomal, gonadal, or anatomical sex is atypical.”

We are aware that not every society member is a practicing clinician working the area of DSD. You can indicate below whether or not you provide care to these patients or if you do not want to participate in the study. By either completing the survey or declining participation, you will not receive any more follow-up reminders to participate.

[2020 only]
Eligibility to participate:
Have you been involved in the clinical management of at least one patient with DSD?

- ☐ Yes
- ☐ No
    -

- Yes [proceed to Enrollment question]
- [if selected “no”]
    - Please complete this last question so we can describe non-participants in any publication.
    - Are you (please select all that apply):
        - In clinical practice, with a different patient population (e.g., diabetes…)
        - In research
        - In teaching
        - In admin
        - Retired
        - Other
            - Other text
    - Thank you for your time

[all years]
Enrollment question:
- ☐ Proceed to Survey
- ☐ Decline Participation

- Yes [proceed to survey]
- [2010, 2020: if selected "decline participation"]
    - So that we can describe non-participants, are you (please select all that apply):
        - In clinical practice with DSD patients
        - In clinical practice, with a different patient population (e.g., diabetes…)
        - In research
        - In teaching
        - In admin
        - Retired
        - Other: [Write-in test response]
    - Thank you for your time

DEMOGRAPHICS

Please describe some characteristics of your practice. The data will be used ONLY for this research study. All responses are strictly confidential. Only averaged data from respondents will be utilized. No information from individual respondents will be provided to any person, group, or agency.

About how many children/adolescents/adults do you see annually who were born with DSD?
[2003: About how many children/adolescents/adults do you see annually who were born with intersexuality?]

_______ cases per year

About how many individuals born with DSD have you seen over your entire career?

_______ cases over entire career

Please indicate the average number of hours per week that you spend in patient care.

______ hours per week

What is your area of specialization?
- ☐ Urology
- ☐ Endocrinology
- ☐ Other: _____

Please describe the community in which your main office/practice is located.
- ☐ Large metropolitan: (Total city and suburban population: 1,000,000 and over)
- ☐ Small metropolitan: (Total city and suburban population: 50,000 - 999,999)
- ☐ Nonmetropolitan/Rural: (Total population: 49,999 or less)

For US Addresses, please indicate the first digit of your office zip code.

____

Where is your practice located?

- ☐ United States
- ☐ Canada
- ☐ Mexico
- ☐ Other

[Note: the following practice location data were not directly collected in 2003, but derived from other items (zip code) + recruitment material and added to the dataset]

[2020: if practice location is United States]
Where is your practice located: State or District (USA)

[2020: if practice location is Canada]
Where is your practice located: Providence or Territory (Canada)

[2020: if practice location is Mexico]
Where is your practice located: State (Mexico)

[2020: if practice location is Other]
Where is your practice located: ______

Primary Practice Setting:

- ☐ Solo or two-physician practice
- ☐ Group practice
- ☐ HMO
- ☐ Medical school or hospital-based
- ☐ Other patient care employment: ____________________________________
- ☐ Other non-patient care employment: ____________________________________ [2010 only]

[if 2003 AND if practice setting – medical school or hospital]
If you are medical school or hospital-based, please indicate the percentage of time devoted to the following in your medical school or hospital-based practice:

% research _____
% patient care _____
% other _____

What is your gender?

- ☐ Male
- ☐ Female
- ☐ Other, specify:___________________

In what year were you born?

___ ___ ___ ___

SURGICAL INFORMED CONSENT

This section concerns your views regarding the ingredients of written informed consent prior to genital surgery in childhood. Please indicate your level of agreement by checking the response option that best represents your view.

To what extent do you agree with the following statements?

Informed consent documents signed by parents prior to genital reconstruction should include the following elements:

	Strongly Disagree	Disagree	Agree	Strongly Agree
There is presently an on-going debate as to whether or not surgery is in the best interest of the child.	☐	☐	☐	☐
Genital abnormalities may take more than one procedure to correct and may in fact involve multiple procedures.	☐	☐	☐	☐
In the future your child may have conflict with their assigned gender and therefore request further surgery to reverse the current surgery.	☐	☐	☐	☐
The alternatives to the recommended surgical procedures have been fully explained to parents and they have been informed that one of these alternatives is to refuse surgical options altogether.	☐	☐	☐	☐

This section concerns current practices:

Do you personally perform genital surgery for DSD?

- ☐ yes [skip to "this section concerns the ingredients…your team uses" items]
- ☐ no

Are you part of a team that offers genital surgeries for DSD?

- ☐ yes
- ☐ no [end section; go to next section]

This section concerns the ingredients of written informed consent that your team uses.

Are the following elements included in the written informed consent that you/your team uses?

	**Yes**	**No**	**I don't know**
There is presently an on-going debate as to whether or not surgery is in the best interest of the child.	☐	☐	☐
Genital abnormalities may take more than one procedure to correct and may in fact involve multiple procedures.	☐	☐	☐
In the future your child may have conflict with their assigned gender and therefore request further surgery to reverse the current surgery.	☐	☐	☐
The alternatives to the recommended surgical procedures have been fully explained to parents and they have been informed that one of these alternatives is to refuse surgical options altogether.	☐	☐	☐

Survey Participants

Recruitment - Procedures
At each timepoint, the research team sought approval from leadership of both the Societies for Pediatric Urology (SPU) and the Pediatric Endocrine Society (PES) to survey their membership and to provide member rosters that include contact information. Leadership of each society approved the research provided rosters, apart from PES at 2020 - citing concerns about burden. As such, for PES in 2020, only those who had previously been invited to participate in either 2003 or 2010 were invited. A publicly available directory PES was reviewed to remove names of those who were no longer listed as PES members; no new members were added.

To account for slightly differing timelines for study approval by SPU and PES and to manage the number of individuals targeted for participation and anticipated follow-up reminders, survey invitations were sent in waves, rather than to all participants at once. Invitation letters that included an explanation of the study and survey login instructions were sent society members. Participants were also offered a paper-and-pencil version upon request. To optimize recruitment, eligible respondents received up to three follow-up requests to participate. After rates of survey completion dropped to minimal levels for several weeks, final requests for participation to non-responders took the form of a phone call and/or a single-page faxed letter encouraging either participation or to otherwise provide a reason for declining to participate in 2003. Through this process, it was learned non-responders were frequently either retired, not in clinical practice, were exclusively involved in research, or not providing care to patients with a DSD. Subsequently, final follow-ups took place via email. Additionally, a screening and opt-out survey was added to the beginning of the 2020 survey to simplify and streamline efforts at identifying participant (in)eligibility and reduce burden associated with follow-ups contacts for non-responders.

Participants were promised confidentially of their responses; procedures were approved by the authors' Institutional Review Boards at the University at Buffalo School of Medicine and Biomedical Sciences (2003) and University of Michigan Medical School (2010 and 2020).

Survey Completeness
In line with research ethics, participants were free to skip over questions they did not wish to answer and discontinue participation at any time. As such, not all surveys have a 100% response rate across all items – the use of branching and skip logic notwithstanding.

Given the order in which major sections of the survey were presented and order of items within sections (see Survey Components, above), discontinuation disproportionately affected response rates of individual items depending on the year of administration.

Eligibility
Common across all timepoints: Current members of either SPU or PES

Table 3. Eligibility requirements unique to specific timepoint:

		2003	2010	2020
Clinical Management	Is currently providing care for patients with intersex/DSD conditions	X	X	X
	Has not provided care for patients with intersex/DSD conditions, but are in a position where they could, potentially, do so now or in the future or otherwise influence clinical care	X		
	Provided care for patients with intersex/DSD conditions in past only[1]	X	X	X
Professional Background	Pediatric Urologist, Other Urologist	X	X	X
	Pediatric Endocrinologist, Other Endocrinologist	X	X	X
	Other professional degree (e.g., PhD)	X		
Practice Location	United States	X	X	X
	Canada	X	X	X
	Mexico	X	X	X
	Other		X	

[1] Emeriti were initially included in the 2003 survey; a combination of difficulty reaching these potential participants due to missing or inaccurate contact information, low participation rates, and feedback from several who targeted for participation indicating that they are retired / unable to provide valid input in their estimation lead to discontinuing recruitment of emeriti as the project progressed.

Participation Rates and Demographics
Given small differences in eligibility criteria for recruitment, participation rates are calculated using the most restrictive eligibility criteria (see Table 3) across study years.

Table 4. Participant ascertainment, recruitment, and participation

	2003		2020	
	SPU	PES	SPU	PES
Names in Directory	263	764	354	494[5]
Ineligible[1]				
Co-I, FG, or PT participant[2]	9	14	8	10
Emeriti	--	117	--	--
Retired	5	11	21	10
Deceased	--	2	--	--
No DSD patients	6	34	37	15
No clinical practice	1	16	3	4
Practice outside North America	--	1	2	8
Other; >1 criteria met	52	53	2	13
Eligible, invited, sample	190	516	281	434
Participated	121 (63.7%)	287 (55.6%)	143 (50.9%)	111 (25.6%)
Began survey without completing[3]	11	13	21	12
Declined participation[4]	10	48	5	5
No Response	48	168	107	304
No contact information	0	0	5	2

[1] Ineligibility was determined at multiple stages. Determinations were made prior to sending survey invitations to members; for others, it occurred after invitations were sent. It is possible that some of those for whom no responses are recorded are ineligible.
[2] Co-investigators (CO-I), focus group (FG) members, and pilot test (PT) participants involved in project design
[3] Participants were free to skip over questions they did not wish to answer and discontinue participation at any time; some completed other portions of the survey without completing any items related to surgical informed consent
[4] A common reason cited for declining participation was being “too busy”
[5] Only current members of PES who had participated in the past were included in the 2020 PES sample; this does not represent the total number of names listed in the PES directory.

Provided they maintained membership in their respective professional society over time, members had the opportunity to participate in up to three waves of the survey. The majority participated on only one occasion; however, within-subjects comparisons are made possible on a limited basis:

Table 5. Participation over time: Surgical informed consent items

Participation Year	Participants (n)		
	SPU	PES	Total (SPU+PES)
2003 only	79	228	307
2020 only	101	52	153
2003 & 2020	42	59	101

Table 6. Participant demographics: Surgical informed consent items

	2003				2020			
	SPU		PES		SPU		PES	
	n	%	n	%	n	%	n	%
Sex								
• Male	115	95.0	172	59.9	122	85.3	58	52.3
• Female	6	5.0	115	40.1	21	14.7	52	46.8
• Other[1]	--	--	--	--	0	0.0	1	0.9
Practice Community[2]								
• Large Metropolitan	86	71.7	173	60.7	107	74.8	76	68.5
• Small Metropolitan	33	27.5	107	37.5	34	23.8	34	30.6
• Nonmetropolitan / Rural	1	0.8	5	1.8	2	1.4	1	0.9
Practice Country								
• United States	114	94.2	269	93.7	137	95.8	103	92.8
• Canada	7	5.8	18	6.3	6	4.2	8	7.2
Practice Setting[3]								
• Medical School or Hospital	78	65.5	212	76.8	104	72.7	94	84.7
• Solo or 2-physician Practice	16	13.4	22	8.0	6	4.2	5	4.5
• Group Practice	24	20.2	33	12.0	30	21.0	10	9.0
• HMO	1	0.8	9	3.3	3	2.1	1	0.9
• Other	0	0.0	0	0.0	0	0.0	1	0.9
	mean	SD	mean	SD	mean	SD	mean	SD
Year of Birth	1951.5	8.9	1950.9	9.1	1965.9	9.4	1960.4	9.2
Cases Seen								
• In Past Year	8.3	8.6	10.1	25.4	11.3	18.0	13.7	20.6
• Over Career	91.3	121.8	65.9	87.8	105.4	166.7	101.8	156.0
Hours/Week in Patient Care	52.6	15.5	28.0	15.6	45.8	13.3	26.2	12.8
Proportion of Time Spent in[4]								
• Research	11.5	11.0	25.0	23.4	--	--	--	--
• Patient Care	77.6	13.8	55.8	25.5	--	--	--	--
• Other	10.9	11.1	19.2	16.6	--	--	--	--

[1] Ineligibility was determined at multiple stages. Determinations were made prior to sending survey invitations to members; for others, it occurred after invitations were sent. It is possible that some of those for whom no responses are recorded are ineligible.
[2] Co-investigators, focus group members, and pilot test participants involved in the design of this project.
[3] Participants were free to skip over questions they did not wish to answer and discontinue participation at any time; some completed other portions of the survey without completing any items related to surgical informed consent
[4] A common reason cited for declining participation was being "too busy."
[5] Only current members of PES who had participated in the past were included in the 2020 PES sample; this does not represent the total number of names listed in the PES directory.
